# Editorial: Towards Expanded Utility of Real Time fMRI Neurofeedback in Clinical Applications

**DOI:** 10.3389/fnhum.2020.606868

**Published:** 2020-11-12

**Authors:** Javier Gonzalez-Castillo, Michal Ramot, Reza Momenan

**Affiliations:** ^1^Section on Functional Imaging Methods, National Institute of Mental Health, National Institutes of Health, Bethesda, MD, United States; ^2^Section on Cognitive Neuropsychology, National Institute of Mental Health, National Institutes of Health, Bethesda, MD, United States; ^3^Department of Neurobiology, Weizmann Institute of Science, Rehovot, Israel; ^4^Clinical NeuroImaging Research Core, National Institute on Alcohol Abuse and Alcoholism (NIAAA), National Institute of Health (NIH), Bethesda, MD, United States

**Keywords:** neurofeeback, fMRI, brain-computer interface (BCI), real-time fMRI (rtfMRI), neuromodulation

Modern functional neuroimaging technologies, such as fMRI and EEG, can stream traces of brain activity in real-time. By presenting those traces back to participants, one can create a closed loop (i.e., neurofeedback loop) that allows individuals to attempt volitional control of their own brain activity. Early neurofeedback research confirmed this hypothetical and showed that participants can indeed volitionally increase and/or decrease levels of activity and connectivity in a diverse set of brain regions with specific outcomes [e.g., somatosensory (Yoo and Jolesz, 2002; Friedrich et al., 2015), having an effect on motor output (Yoo and Jolesz, 2002), or social interaction in children with Autism Spectrum Disorder (Friedrich et al., 2015); amygdala (Zotev et al., 2011; Young et al., 2017; Keynan et al., 2019) in healthy participants (Zotev et al., 2011) or for patient treatment (Young et al., 2017; Keynan et al., 2019); posterior cingulate cortex (Garrison et al., 2013; Brewer and Garrison, 2014) for recording (Garrison et al., 2013) and augmenting (Brewer and Garrison, 2014) mindfulness effect of medication]. In this sense, neurofeedback can be regarded as a minimally-invasive form of neuoromodulation that does not require any surgical intervention, unlike neuromodulation methods such as deep brain stimulation (DBS) procedures (Lozano et al., 2019). It also does not require other forms of external intervention, such as the injection of strong magnetic fields as with transcranial magnetic stimulation (TMS) (Valero-Cabre et al., 2017), or electrical currents as in transcranial direct current stimulation (tDCS) (Zhao et al., 2017). Importantly, despite this lack of invasiveness, and therefore overt risk for the patient, repeated practice with neurofeedback can produce stable long-term changes in behavior (Shibata et al., 2011; Amano et al., 2016; Ramot et al., 2017), suggesting neurofeedback could become an alternative/complementary therapeutic approach in situations where more traditional methods (e.g., pharmacological and/or behavioral) do not suffice.

As mental healthcare struggles with finding effective therapies, we believed it was important to review the current state-of-the-art of neurofeedback research for clinical applications. More particularly, we decided to focus on fMRI-based neurofeedback given its greater ability to accurately target specific brain regions and to reach deeper structures (e.g., amygdala). Yet, it is worth noticing that other modalities, such as EEG (Loo and Makeig, 2012; Simkin et al., 2014; Marzbani et al., 2016; Wang et al., 2019) and fNIRS-based neurofeedback (Kohl et al., 2020), also hold great potential for clinical use, as well as their combined use [see (Lioi et al., 2020) in this issue for EEG-fMRI combination].

This special issue contains seven articles that explore the clinical utility of fMRI-based neurofeedback in diverse populations [e.g., autism (Pereira et al.), stroke (Lioi et al.; Mehler et al.), depression (Quevedo et al.)], targeting different brain regions [e.g., somatosensory (Kaas et al.), fusiform face area (Pereira et al.), or whole-brain based classification (Bagarinao et al.)], and using quite distinct neurofeedback setups, procedures, and analytical tools. It also includes a review (Fede et al.) aimed at helping make informed decisions in the design of future neurofeedback studies. The diversity of this work speaks not only to the infancy of the field—which manifests in lack of standardized ways to practice fMRI-based neurofeedback—but also to the potential for creativity and the manifold opportunities that neurofeedback holds for mental healthcare. For example, Pereira et al. explore how neurofeedback of the fusiform face area (FFA) may help people with autistic spectrum disorder improve their ability to process human faces, which has been previously attributed to hypoactivation of this region. The authors show how neurofeedback was accompanied by changes in connectivity across regions of the ventral visual stream (of which the FFA is part), among other significant changes. Similarly, Lioi et al. explore the ability of fMRI-neurofeedback to improve rehabilitation outcomes in stroke patients. For this purpose, the authors propose an innovative bimodal (e.g., EEG and fMRI) neurofeedback approach that targets ipsilateral primary cortex, and demonstrate, in at least two patients, a functional improvement of upper limb motricity. The potential of fMRI-neurofeedback to improve rehabilitative outcomes in stroke patients is also examined by Mehler et al.. In particular, they focus on the ability of graded feedback applied to supplementary motor cortex. Their findings were mostly negative, which by no means undermines the importance of the study. On the contrary, by following pre-registration procedures, the authors demonstrate the importance of rigorous hypothesis testing in these early stages of the field, as well as the difficulty to apply current pre-registration frameworks to proof-of-concept neurofeedback studies. Finally, Quevedo et al. describe an investigation of how neurofeedback may provide an alternative treatment for adolescent depression. In this case, participants were instructed to attempt upregulation of limbic activity via recall of positive autobiographical memories. The authors were able to identify changes in limbic connectivity, as well as short-term changes in depression and rumination scores, following neurofeedback. Together, these four studies highlight the great potential for clinical applications of neurofeedback, yet also tell us a cautionary tale about the need for larger scale studies and the need to better understand inter-subject variability of neurofeedback interventions.

In addition to these “*direct*” clinical applications, this issue also includes a series of studies that focus primarily on methodological development for fMRI-Neurofeedback. First, Bagarinao et al. investigate how learning effects modulates performance of brain state classifiers used in neurofeedback. In particular, they show that, as training progresses, activity patterns decrease in extent, which negatively affects classifiers accuracy. They propose the continuous update of classifiers after each neurofeedback run as a way to account for this undesired phenomenon. Next, Kaas et al. describe how fMRI-neurofeedback can help optimize brain-computer interface protocols currently under development to help patients with locked-in-syndrome communicate with the external world. Their article explains recent work looking at how the higher sensitivity of ultra-high imaging systems (i.e., 7T scanners) can be leveraged to distinguish between different patterns of somatosensory imagery at the level of individual-trials, and how such protocols could be used for binary communication (responding to yes/no questions). Finally, this issue concludes with a review article by Fede et al. looking at building consensus about best practices for the field. The authors reviewed 146 published neurofeedback studies and looked at differences in sample size, strategy, timing, control conditions and number of sessions, among many other experimental factors. Based on their analyses, the authors provide a series of suggestions for how to optimally design future neurofeedback studies.

It is our hope that this collection of work will bring excitement to the broader neuroscience community about the clinical potential of fMRI-based neurofeedback. We also hope it will help ignite avid discussions about how to move the field forward. For example, readers will often find the term “proof-of-concept” in the abstracts of these manuscripts, as well as great variability in outcome metrics. This highlights the need for bigger studies enrolling larger numbers of participants. It also highlights the need to better understand inter-subject variability in terms of effectiveness and “*dosage*.” It may be that not all people can volitionally control their brain activity, or that we all can, but we may need different amounts of training and guidance (i.e., “*dosage*”) while doing so (as is the case with many other aspects of learning). Similarly, other pressing issues for the field include understanding what are the optimal spatial and temporal resolutions for neurofeedback, as well as their limits. For example, could individuals control activity at the laminar-level? The field is also lacking in terms of standardization of protocols and outcome measures, so that studies can be combined in order to augment statistical power. Also, for fMRI-neurofeedback to be considered a viable alternative to existing traditional interventions, researchers should perform comparative studies to evaluate how neurofeedback relates to other existing alternatives (e.g., behavioral, pharmacological) in terms of effectiveness, early termination of treatment, severity of side effects and monetary cost. As with any incipient field, the questions are endless and so are the opportunities. Fortunately for fMRI-based neurofeedback, the community is finding ways to navigate the unknowns and build a theory and practice that may soon provide additional hope for those afflicted by debilitating mental health conditions.

## Author Contributions

JG-C, MR, and RM contributed to the writing and editing of this article. All authors contributed to the article and approved the submitted version.

## Conflict of Interest

The authors declare that the research was conducted in the absence of any commercial or financial relationships that could be construed as a potential conflict of interest.
